# Local IgG autoantibody profiles in hidradenitis suppurativa and their associations with disease features and anti-TNF therapy

**DOI:** 10.1016/j.xjidi.2026.100467

**Published:** 2026-03-18

**Authors:** Carmelo Carmona-Rivera, Liam J. O’Neil, Teja Mallela, Christopher J. Sayed, Mariana J. Kaplan

**Affiliations:** 1 Systemic Autoimmunity Branch, National Institute of Arthritis and Musculoskeletal and Skin Diseases, National Institutes of Health, Bethesda, Maryland, USA; 2 Manitoba Centre for Proteomics and Systems Biology, Department of Internal Medicine, University of Manitoba, Winnipeg, Manitoba, Canada; 3 Department of Dermatology, University of North Carolina, Chapel Hill, North Carolina, USA

**Keywords:** Adaptive immunity, Anti-TNF therapies, Autoantibodies, Hidradenitis suppurativa, IgG

## Abstract

Hidradenitis suppurativa (HS) is a chronic inflammatory skin disorder marked by dysregulated TNF-α signaling and aberrant adaptive immune responses, yet the role of humoral autoimmunity in disease pathogenesis remains incompletely understood. Although autoantibodies have been reported in HS, their prevalence, specificity, and clinical relevance, particularly in relation to clinical features and biologic therapy, are not well defined. This study aimed to characterize lesional IgG autoantibody profiles in HS and evaluate their associations with clinical features, comorbidities, and biologic treatment status. Skin biopsy specimens from 20 patients with HS and 5 control subjects were analyzed for IgG autoantibodies, and findings were correlated with clinical features. Patients with HS demonstrated significantly elevated lesional IgG autoantibody levels, with greater abundance observed in chronic disease stages. Higher autoantibody levels were more prevalent among Black patients and correlated with disease severity (Hurley stage, hidradenitis suppurativa lesion, area, and severity index), lesion type (sinus tracts, scarring), comorbidities including asthma, acne, and diabetes, and clinical factors such as pain severity and smoking status. Samples from patients receiving adalimumab showed lower autoantibody levels, whereas no consistent pattern was observed with infliximab. These findings identify lesional IgG autoantibodies as a feature of HS and support a role for humoral autoimmunity in HS pathobiology, warranting longitudinal investigation.

## Introduction

Hidradenitis suppurativa (HS) is a chronic, relapsing inflammatory skin disorder that primarily affects intertriginous areas, such as the axillae, groin, and perianal regions (Jemec and Kimball, 2015; Mintoff and Pace, 2024; van Straalen et al, 2022). Clinically, HS is characterized by painful nodules, abscesses, tunnels, and scarring that contribute to significant physical and psychological morbidity (Mintoff and Pace, 2024; van Straalen et al, 2022).

The pathogenesis of HS is multifactorial, involving follicular occlusion, dysregulated immune responses, and aberrant tissue remodeling (Byrd et al, 2019; Frew et al, 2019, 2018; Zouboulis et al, 2020a, 2020b). Among the key inflammatory mediators, TNF-α is considered a central driver of disease activity (Frew et al, 2018). Elevated levels of TNF-α have been consistently observed in both the serum and lesional skin of patients with HS compared to healthy individuals (Matusiak et al, 2009; van der Zee et al, 2011). This is further supported by the clinical efficacy of anti-TNF therapies, highlighting the critical role of TNF-α–mediated inflammation in HS progression (Alikhan et al, 2019; Jemec et al, 2019; Kimball et al, 2018, 2016; Lowe et al, 2020; Zouboulis et al, 2019).

In addition to TNF-driven pathways, there is growing evidence that adaptive immune mechanisms, particularly humoral immunity, play a significant role in HS pathophysiology (Byrd et al, 2019; Gudjonsson et al, 2020; Lowe et al, 2024; Oliveira et al, 2023). IgG autoantibodies have been identified in the lesional skin of patients with HS, often in conjunction with the formation of tertiary lymphoid structures (TLS) (Carmona-Rivera et al, 2022; Gudjonsson et al, 2020; Lowe et al, 2024; Oliveira et al, 2023; Yu et al, 2024). These ectopic lymphoid aggregates facilitate local antigen presentation and autoantibody production, suggesting an active and organized autoimmune component.

We have previously demonstrated a link between the presence of IgG autoantibodies and inflammation within skin lesions, further implicating these autoantibodies in disease processes (Carmona-Rivera et al, 2022; Oliveira et al, 2023).

Despite these advances in understanding, a critical knowledge gap remains regarding the clinical significance of IgG autoantibodies in HS. Although their presence has been associated with disease severity and local immune activation (Carmona-Rivera et al, 2022), it is still unclear whether these autoantibodies contribute to other clinical manifestations or comorbidities commonly seen in HS. Furthermore, their potential utility as biomarkers for predicting disease progression or treatment response has yet to be determined.

## Results

### Anti-TNF therapies exhibit distinct impacts on IgG autoantibody levels

**Figure 1:**
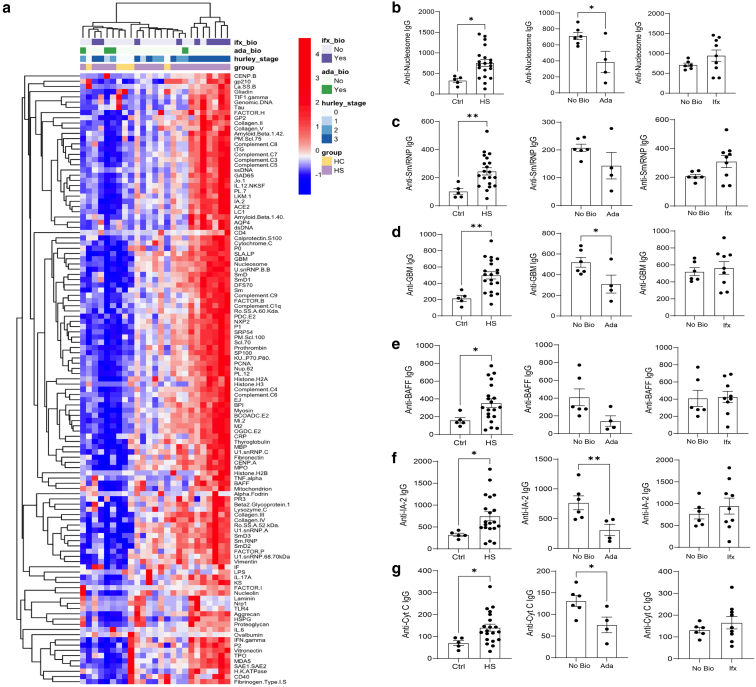
**Adalimumab treatment decreases IgG autoantibodies in HS skin lesions.** (**a**) Unsupervised clustering heatmaps display IgG autoantibody profiles in skin tissue from healthy controls (n = 5) and HS patients (n = 20). **(b)** Skin lysates were analyzed for IgG autoantibodies targeting nucleosomes. **(c)** SmRNP. **(d)** GBM. **(e)** BAFF. **(f)** IA-2. **(g)** cytochrome C. Comparisons were made between patients with HS not receiving biologic therapy (no bio, n = 6) and those treated with either ada (n = 4, b–g middle panels) or Ifx (n = 9, b–g right panels). Data are presented as mean ± SEM. Statistical significance was assessed using the Mann-Whitney U test; ∗*P* < .05 and ∗∗*P* < .01. Ada: adalimumab; BAFF, B-cell activating factor; GBM, glomerular basement membrane; HS, hidradenitis suppurativa; IA-2, islet antigen-2; Ifx, infliximab; SmRNP, Smith/ribonucleoprotein complex.

To investigate the presence of IgG autoantibodies in HS skin lesions, skin samples from twenty patients with HS and five healthy volunteers were pulverized in liquid nitrogen using a mortar and pestle, as described in the Materials and Methods section. The demographic and clinical characteristics of the study cohorts are summarized in Table 1. Proteins were extracted from the samples and analyzed using a comprehensive panel of antigens commonly associated with established rheumatic diseases. A Cy3-labeled anti-human assay revealed elevated levels of IgG autoantibodies targeting self-antigens in HS skin lesions (Figure 1a).

**Table 1:** Demographics of the Patients with HS Tested in This Study

Demographic	HS (n = 20)
Age, (IQR) y	34 (12)
Female sex, n (%)	14 (70)
Race	
Black n (%)	10 (50)
White n (%)	8 (40)
No black or white n (%)	2 (10)
History	
Smoking	
Current use, n (%)	1 (5)
Previous smoker, n (%)	3 (15)
Never smoked, n (%)	16 (80)
BMI (IQR)	35 (13)
Diabetes, n (%)	5 (25)
Draining sinus, n (%)	8 (40)
Intertriginous comedones, n (%)	6 (30)
Nodule, n (%)	17 (85)
Non-draining sinus, n (%)	6 (30)
Scar tissue, n (%)	2 (10)
Treatment	
Adalimumab, n (%)	4 (20)
Infliximab, n (%)	9 (45)
Topical clindamycin, n (%)	6 (30)

Abbreviations: BMI, body mass index; HS, hidradenitis suppurativa; IQR, interquartile range.

Unsupervised cluster analysis indicated that these autoantibodies were predominantly found in the chronic stages of the disease (Figure 1a). Specifically, IgG antibodies against nucleosomes (Figure 1b), Smith/ribonucleoprotein complex (SmRNP; Figure 1c), glomerular basement membrane (GBM; Figure 1d), B-cell activating factor (BAFF; Figure 1e), islet antigen-2 (IA-2; Figure 1f), and cytochrome C (Figure 1g) were significantly elevated in patients with HS compared to healthy controls, consistent with our previous findings (Carmona-Rivera et al, 2022).

Given that anti-TNF therapies are commonly used as first-line treatments for HS (Alikhan et al, 2019; Jemec et al, 2019; Kimball et al, 2018), we next evaluated lesional IgG autoantibody profiles in patients receiving adalimumab (Ada) or infliximab (Ifx) at the time of biopsy. In this cross-sectional analysis, samples from patients treated with Ada tended to exhibit lower IgG autoreactivity against nucleosomes, SmRNP, GBM, BAFF, IA-2, and cytochrome C compared with samples from patients not receiving biologic therapy (Figure 1b–g, middle panels). In contrast, no consistent pattern was observed among patients treated with Ifx (Figure 1b–g, right panels). These findings are descriptive and do not establish causal effects of therapy; observed differences may reflect baseline disease characteristics, treatment selection, disease duration, or other confounding factors rather than direct effects of the biologic agents themselves.

### IgG autoantibodies correlate with clinical manifestations in hidradenitis suppurativa

To investigate the clinical relevance of autoantibodies involved in HS pathogenesis, patients underwent a comprehensive and detailed clinical evaluation. Data collected included disease severity measures such as the hidradenitis suppurativa lesion, area, and severity index score, global assessment, and the international hidradenitis suppurativa severity score system. Lesion-specific characteristics were recorded, including the number of nodules, draining and non-draining tunnels, presence of scars, pilonidal cysts, intertriginous epidermoid cysts, and comedones.

Additional patient-reported and clinical factors were assessed, including the presence of pain, self-reported disease severity, vitamin D deficiency, use of topical clindamycin, and comorbidities such as hypertension, allergies, depression, asthma, and acne. Demographic and treatment-related variables were also documented, including race, body mass index, Hurley stage, and current treatment with either Ada or Ifx.

**Figure 2:**
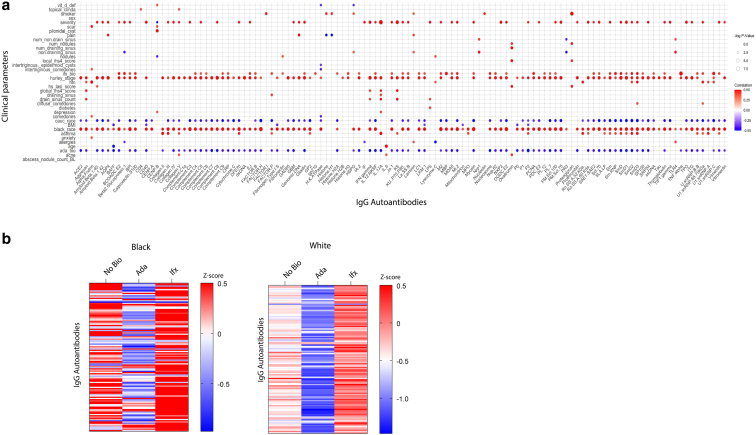
**Levels of skin IgG autoantibodies correlate with clinical manifestations.** (**a**) Correlation analysis of IgA autoantibody levels in HS skin lesions with clinical outcomes (n = 20), with the color gradient indicating correlation coefficients (r values). Ada: adalimumab;. All intersections displayed in this correlome are significant, ∗*P* < .05. **(b)** Heatmaps show IgG autoantibody profiles stratified by race among patients with HS not receiving biologic therapy (no bio) and those treated with ada or Ifx. HS, hidradenitis suppurativa. Ada, adalimumab; htn: hypertension; Ifx: infliximab; pain: numeric pain rating score; severity: self-reported severity.

IgG autoantibodies were significantly more prevalent in Black patients. Of the 120 autoantibodies tested, 85% showed significant association with Black race, including strong correlations with anti-Factor H (r = 0.7209, *P* = .0001), anti-Myosin (r = 0.7030, *P* = .0002), and anti-BPI (r = 0.6888, *P* = .0004), highlighting substantial racial variation in humoral immune responses in HS (Figure 2a). In contrast, 66% of autoantibodies were negatively associated with White patients, with strong inverse correlations observed for anti-Factor H (r = −0.6017, *P* = .0025), anti-SmD2 (r = −0.5799, *P* = .0036), and anti-SmRNP (r = −0.5313, *P* = .0079) (Figure 2a).

Disease severity also showed broad associations with autoantibody profiles. Approximately 75% of autoantibodies correlated with Hurley stage, with top associations seen for anti-Complement C8 (r = 0.6726, *P* = .0005), anti-SmD (r = 0.6632, *P* = .0007), and anti-IFN-γ (r = 0.6495, *P* = .0009) (Figure 2a). Similarly, hidradenitis suppurativa lesion, area, and severity index and international hidradenitis suppurativa severity score system scores were significantly linked to autoantibodies, including anti-ovalbumin, anti-proteoglycan, anti-amyloid beta 1–40, anti-IL-17A, and anti-Aggrecan (Figure 2a, Supplementary Table S1), suggesting autoantibodies reflect clinical burden.

Lesion morphology and distribution further aligned with specific antibody profiles. Draining tunnel counts were significantly associated with anti-KS (r = 0.5386, *P* = .0156), anti-IL-17A (r = 0.4841, *P* = .0287), and anti-aggrecan (r = 0.4641, *P* = .0350), while non-draining sinuses correlated with anti-NRP1 (r = 0.4926, *P* = .0136), and anti-toll-like receptor 4 (r = 0.4774, *P* = .0166). Comedones and their distribution (diffuse, intertriginous) were significantly linked to antibodies against alpha fodrin, SmD3, and U1 snRNP 68 kDa, with anti-H/K ATPase (r = –0.4200, *P* = .0326) showing a negative correlation in intertriginous areas (Figure 2a, Supplementary Table S1). Scars and pilonidal cysts were significantly associated with anti-alpha fodrin (r = 0.4878, *P* = .0200) and anti-CENP B (r = 0.5885, *P* = .0031) (Figure 2a), respectively.

Several comorbidities and symptoms were linked to distinct autoantibody signatures. Body mass index was negatively associated with antibodies such as anti-BAFF (r = −0.4486, *P* = .0236), anti-fibrinogen (r = −0.4189, *P* = .0329), and anti-CD40 (r = −0.4170, *P* = .0336). Diabetes was significantly associated with anti-lipopolysaccharide (r = 0.4295, *P* = .0293), while pain correlated positively with anti-genomic DNA, AQP4, and laminin, and negatively with anti-histone H1(r = −0.5335, *P* = .0166) (Figure 2a, Supplementary Table S1). Acne was associated with antibodies against ovalbumin, collagen V, and IL-6 (Figure 2a, Supplementary Table S1).

Autoantibodies also reflected broader immune activation in patients with asthma, showing significant associations with anti-IL-17A, collagen II, SmD3, and U1 snRNP A (Figure 2a, Supplementary Table S1). Smoking was significantly associated with elevated levels of anti-proteoglycan, factor I, histone H1, nucleolin, and laminin antibodies (Figure 2a, Supplementary Table S1), underscoring potential environmental influences on autoimmunity. No significant associations were observed with sex.

At this cross-sectional time point, treatment with Ada was broadly associated with reduced autoantibody responses, with 72% of tested autoantibodies showing significant negative associations (Figure 2a). In contrast, Ifx treatment was positively associated with many of the same autoantibodies. To determine whether these associations between anti-TNF therapy and autoantibody levels were confounded by race, we generated a heatmap summarizing autoantibody trends stratified by race group. Across both Black and White patients, Ada treatment was consistently associated with lower autoantibody levels, indicating that the observed treatment-associated differences were not driven by ethnicity (Figure 2b). These data highlight treatment-associated differences in lesional IgG autoantibody profiles in HS; however, such differences may reflect underlying variation in patient characteristics, disease severity, or treatment history rather than direct effects of the therapies themselves.

## Discussion

This study identifies distinct IgG autoantibody profiles in HS lesional skin. IgG autoreactivity, particularly against nucleosomes, SmRNP, GBM, BAFF, IA-2, cytochrome C, and others, was elevated in HS samples, with higher levels observed in more chronic or severe disease stages. These findings indicate the presence of adaptive immune activation and local autoantibody accumulation in HS, without establishing a causal role for these antibodies in disease pathogenesis (Byrd et al, 2019; Frew et al, 2020; Gudjonsson et al, 2020; Lowe et al, 2024; Yu et al, 2024).

Notably, Ada, the only FDA-approved TNF-α inhibitor for moderate-to-severe HS (Alikhan et al, 2019; Jemec et al, 2019; Kimball et al, 2016; Zouboulis et al, 2019), was associated with a significant reduction in most of these disease-associated autoantibodies. In contrast, Ifx is associated with increased autoantibody levels. These divergent responses suggest that not all anti-TNF-α therapies modulate adaptive immunity in HS equivalently. Ada is a fully human monoclonal IgG1 antibody, whereas Ifx is a chimeric mouse-human monoclonal antibody (Louis et al, 2003; Shih et al, 2022). The immunogenic nature of Ifx, which can elicit anti-drug antibodies and immune complex formation, may underline the paradoxical increase in IgG autoantibodies observed in some patients (Louis et al, 2003; Vaz et al, 2016). Additionally, patients receiving Ifx often represent a more treatment-refractory population, which may further explain the strong associations between autoantibody levels and treatment.

Mechanistically, these findings are consistent with emerging data on TLS in HS lesions. TLS facilitate local antigen presentation and autoantibody production, and their development is promoted by TNF-α–regulated chemokines, such as CXCL13 and CCL19 (Lowe et al, 2024; Yu et al, 2024). Although experimental studies suggest that TNF-α inhibition may influence TLS formation and humoral immunity, the cross-sectional design of the present study precludes direct assessment of these mechanisms. Longitudinal and mechanistic studies will be required to determine whether modulation of TLS contributes to changes in local autoantibody profiles in HS.

Our study also highlights strong correlations between autoantibody profiles and clinical parameters, including disease severity (eg, Hurley stage, international hidradenitis suppurativa severity score system, hidradenitis suppurativa lesion, area, and severity index), lesion morphology (eg, sinus tracts, scarring, comedones), and comorbid conditions such as asthma, acne, and pain. Additionally, significant racial differences in autoantibody expression were observed, with a greater prevalence and diversity in Black patients, suggesting the need for personalized approaches to immune profiling and treatment in HS.

This study suggests that elevated IgG autoantibody levels in HS lesions likely reflect cumulative tissue damage, disease chronicity, and local inflammatory burden rather than a primary pathogenic driver. The observed modulation of autoantibody levels in patients receiving effective anti-inflammatory therapy underscores the dynamic nature of these humoral responses and supports their potential utility as biomarkers of disease activity and immune dysregulation. Importantly, the reduction in lesional IgG autoreactivity observed in samples from patients treated with Ada may reflect broad suppression of inflammation rather than a TNF-specific effect, a distinction that will require comparative studies with other targeted therapies. Moreover, although HS-specificity cannot be inferred from the present data, the autoantibody patterns identified may reflect shared inflammatory pathways across IL-17 and TNF-driven skin diseases, highlighting the need for cross-disease investigations.

Several limitations should be considered. First, analyses were limited to lesional skin biopsies, and serum samples were not available; evaluation of circulating autoantibodies will be necessary to assess their potential as non-invasive systemic biomarkers. Second, the cross-sectional design precludes assessment of longitudinal dynamics, baseline-to-post-treatment changes, or causal relationships between autoantibody modulation and therapy. Differences observed across treatment groups may therefore reflect underlying patient characteristics, disease severity, or prior treatment history, rather than direct drug effects. Third, the modest sample size limited statistical power to fully evaluate associations with clinical features, comorbidities, smoking status, and racial differences, and may have contributed to variability in observed autoantibody profiles. Finally, the study cannot distinguish TNF-specific mechanisms from general consequences of inflammation suppression, highlighting the need for larger, longitudinal, and mechanistic studies.

To our knowledge, this study systematically profiles local IgG autoantibodies in HS and relates them to clinical parameters. These findings provide, to our knowledge, previously unreported insights into the contribution of humoral immune activation within the inflammatory milieu of HS and generate hypotheses regarding whether specific autoantibodies serve as biomarkers, participate in disease mechanisms, or represent bystander phenomena. At this cross-sectional time point, Ada-treated samples tended to show lower IgG autoantibody levels in lesional skin compared with untreated samples, consistent with its established clinical efficacy and FDA-approved status for moderate-to-severe HS (Alikhan et al, 2019; Jemec et al, 2019; Kimball et al, 2018, 2016; Zouboulis et al, 2019). Although mechanisms remain speculative, these differences may reflect modulation of local TLS and B cell activity (Yu et al, 2024). The observed associations between autoantibodies and clinical features suggest that profiling humoral responses could inform future studies on disease stratification and therapeutic monitoring. Longitudinal studies incorporating paired tissue and serum samples will be essential to define the temporal dynamics, biological relevance, and clinical utility of IgG autoantibody signatures in HS, and to determine their value for disease stratification and therapeutic monitoring.

## Materials and Methods

### Tissue sample collection

Skin biopsies were collected during routine surgical procedures using a 6-mm punch. Clinical assessments were performed by the study investigator at the time of surgery and included lesion type, regional and global lesion counts, current medications, and other relevant clinical or demographic information obtained from electronic medical records or clinical registries. Race and ethnicity information for participants were obtained from the electronic health record, as HS disproportionately affects Black populations and race and ethnicity are therefore important demographic variables to capture in studies of this condition. All HS diagnoses were confirmed via clinical, visual, and histopathological evaluation. Collected tissues were immediately stored at –80 °C until further processing.

### Protein isolation from tissue

Protein extraction was conducted as previously described (Byrd et al, 2019; Oliveira et al, 2023). Briefly, frozen tissue samples were pulverized and resuspended in RIPA buffer supplemented with a protease inhibitor cocktail (Roche). After 1-hour incubation at 4 °C, lysates were centrifuged at 14,000 r.p.m for 10 minutes. Supernatants were collected and transferred to Eppendorf tubes. Protein concentrations were measured using the BCA Protein Assay Kit (Thermo Fisher Scientific), following the manufacturer’s protocol.

### IgG autoantibody detection assay

IgG autoantibody profiling was performed as described in prior studies (Carmona-Rivera et al, 2022; Lammert et al, 2020). Skin lysates from 20 patients with HS and 5 healthy controls were pretreated with DNase I and diluted 1:50. Samples were incubated on nitrocellulose membrane-coated slides printed in duplicate with a curated panel of autoantigens associated with autoimmune diseases (available at: https://utsw.corefacilities.org/service_center/show_external/5596?name=utsw-microarray-immune-phenotyping-core-facility). After hybridization and washing, bound IgG antibodies were detected using Cy3-labeled anti-human IgA (1:2000; Jackson ImmunoResearch Laboratories). Arrays were scanned using a GenePix 4400A Microarray Scanner, and fluorescence intensities were quantified using GenePix 7.0 software (Molecular Devices). Background signal (PBS) was subtracted from each sample’s average signal intensity. A signal-to-noise ratio was calculated, and antibody scores were determined using the formula log_2_ (SNR × NFI + 1), as previously reported (Perez-Diez et al, 2020).

### Statistical analysis

All statistical analyses were performed using GraphPad Prism version 9.5.0 (GraphPad Software, La Jolla, CA, USA). For comparisons involving non-normally distributed data, the Mann–Whitney U test was used. Pearson correlation coefficients were calculated to assess relationships between continuous variables. A *P*-value < .05 was considered statistically significant.

## Ethics Statement

Hidradenitis suppurativa and control skin samples were collected under the institutional review board protocol #IRB-17-1506, approved by the University of North Carolina institutional review board. Lesional skin from patients with hidradenitis suppurativa was obtained through the University of North Carolina Department of Dermatology’s hidradenitis suppurativa program for clinical and research excellence (ProCARE), following written informed consent.

## Data Availability Statement

Datasets generated from this study have been provided in the Supplementary Materials.

## ORCIDs

Carmelo Carmona-Rivera: http://orcid.org/0000-0002-7706-1464

Liam J. O’Neil: http://orcid.org/0000-0001-5580-6472

Christopher J. Sayed https://orcid.org/0000-0003-3201-4637

Mariana J. Kaplan: http://orcid.org/0000-0003-2968-0815

## Conflict of Interest

CJS serves as secretary of the Board of Directors for HS Foundation, speaker for AbbVie, Novartis and UCB, consultant for AbbVie, Novartis, UCB, Sanofi, Incyte, InfaRx, AstraZeneca, Navigator Medicines, Moonlake Therapeutics and Elasmogen, and an investigator for Novartis, UCB, Incyte, InflaRx and AstraZeneca. All other authors state no conflict of interest.

## Supplementary Material

Supplementary Table S1 XXX
